# Recent advances in research on high-frequency mutations in the ATP7B gene associated with Wilson disease in the Chinese population: from genetic evolution to precision medicine

**DOI:** 10.3389/fmolb.2026.1873468

**Published:** 2026-07-31

**Authors:** Qiaoyu Xuan, Daiping Hua, Lanting Sun, Qiyan Ma, Wenming Yang, Han Wang

**Affiliations:** 1 Department of Neurology, The First Affiliated Hospital of Anhui University of Chinese Medicine, Hefei, China; 2 Key Laboratory of Xin’An Medicine, Ministry of Education, Anhui University of Chinese Medicine, Hefei, China

**Keywords:** ATP7B, P992L mutation of ATP7B, precision medicine, R778L mutation of ATP7B, Wilson disease

## Abstract

Wilson disease (WD) is a hereditary disorder of copper metabolism caused by mutations in the ATP7B gene; the Chinese population exhibits a unique, high-frequency mutation profile centered on the R778L and P992L mutations. This article provides a narrative review of the genetic evolution, molecular pathogenic mechanisms, phenotypic heterogeneity, and precision diagnosis and treatment strategies for high-frequency ATP7B mutations in the Chinese WD population. The high-frequency enrichment of R778L and P992L is speculated to arise from the combined effects of CpG mutation hotspots and the founder effect supported by East Asian haplotype data, with distinct geographical distributions and relatively conserved haplotypes observed in existing cohorts. Functional studies indicate that the R778L variant disrupts the transmembrane domain and induces severe protein misfolding associated with an early-onset hepatic phenotype, whereas the P992L mutation partially retains catalytic function and tends to present neurological manifestations in many clinical cohorts, though individual phenotypic variation remains substantial. Preclinical molecular chaperone and gene editing strategies offer potential mechanistic targets for mutation-specific intervention, yet their clinical translation remains at an immature stage with insufficient human trial data. In the future, it will be necessary to establish multicenter cohorts, advance mutation-targeted therapies, and develop a precision diagnosis and treatment system covering the entire disease lifecycle.

## 1 Introduction

Wilson disease (WD) is an autosomal recessive disorder of copper metabolism caused by mutations in the *ATP7B* gene. The core pathological mechanism involves a functional defect in the ATP7B protein, which impairs the excretion of copper via bile. This leads to abnormal copper deposition in multiple organs, including the liver, central nervous system, and cornea, resulting in progressive, irreversible tissue damage and multisystem dysfunction (Członkowska et al., 2018; Schilsky et al., 2023). The *ATP7B* gene encodes a P-type ATPase involved in copper transport, which maintains copper homeostasis by participating in ceruloplasmin synthesis and mediating the excretion of copper in bile (Bitter et al., 2022; Piotr et al., 2025).

As one of the most common inherited liver diseases worldwide, the prevalence and mutation spectrum of WD exhibit significant racial and geographical heterogeneity (Beyzaei et al., 2024; Ferenci et al., 2003; Ferenci, 2006). Globally, WD prevalence ranges from 1 in 50,000 to 1 in 30,000 (Sandahl et al., 2020), while the Chinese population carries a higher estimated prevalence of 5.87 per 100,000 (Xie and Wu, 2017). Caucasian populations are dominated by the H1069 variant (40%–60% of pathogenic alleles) (Ferenci, 2006; Ferenci et al., 2019), whereas Chinese patients feature a unique mutational landscape defined by R778L and P992L, which together account for over 40% of disease-causing alleles and display distinct geographic spread patterns (Zhang et al., 2022; Xia et al., 2024; Fan et al., 2023; Li et al., 2013). These divergent mutational backgrounds translate to varied ages of onset, organ involvement, copper biochemistry profiles, drug responses and prognostic risks among Chinese WD patients, meaning Western clinical guidelines cannot be directly extrapolated to East Asian cohorts. A dedicated narrative review of these two hotspot mutations therefore carries unique clinical value for building region-specific precision diagnostic, therapeutic and genetic screening frameworks.

In recent years, with advances in molecular genetics and high-throughput sequencing technologies, Chinese researchers have made a series of breakthroughs regarding the distribution patterns of high-frequency mutations in WD, its molecular pathogenic mechanisms, and genotype–phenotype associations (Li et al., 2019; Zhang et al., 2024; Huang et al., 2022). Nonetheless, major knowledge gaps persist: the population genetic origins of R778L and P992L remain poorly defined; mechanisms underlying phenotypic heterogeneity are incompletely understood; modulatory pathways involving modifier genes, inflammation and non-coding RNAs require systematic exploration; and mutation-targeted precision interventions are still confined to preclinical testing rather than routine clinical application (Shribman et al., 2021; Zeng et al., 2025).

This narrative review synthesizes existing evidence across four core dimensions—population genetic evolution, molecular pathogenic mechanisms, clinical phenotypic characteristics and targeted intervention—to refine WD pathogenesis theories and support the shift from empirical management to genotype-guided precision care for Chinese patients. We summarize current research progress, unresolved limitations and prospective research directions to inform future basic and clinical investigations.

## 2 The genetic and evolutionary basis of high-frequency *ATP7B* mutations in the Chinese population

The high prevalence of the R778L and P992L mutations in the Chinese WD population is hypothesized to stem from the joint influence of recurrent CpG mutagenesis, genetic drift and potential founder effects inferred from haplotype linkage data across East Asian cohorts. Furthermore, the distinct spatiotemporal distributions and haplotype characteristics exhibited by these two factors constitute a core feature of the genetic background of the Chinese WD population (Zhang et al., 2022; Dong et al., 2016; Yang et al., 2021).

### 2.1 Differentiated patterns of spatiotemporal distribution

R778L is the most common mutation among Han Chinese patients with WD, with an allele frequency of approximately 30% (Zhang et al., 2022). It distributes uniformly nationwide and has been identified in multiple ethnic minorities, pointing to an ancient, shared Han ancestral origin that spread via historical migration across East Asia (Dong et al., 2016; Yang et al., 2021; Wang et al., 2023; Mak et al., 2008). In contrast, P992L follows a clear north–south frequency gradient: it is far more common south of the Yangtze River. This variant arose in southern Han ancestral groups and established local founder enrichment; subsequent northward population migration diluted its allele frequency with increasing latitude (Zhang et al., 2022; Li et al., 2019; Zhang et al., 2024; Yang et al., 2021; Jia et al., 2022).

A cross-sectional comparison of mutation profiles in East Asian populations further supports the unique genetic evolutionary characteristics of ATP7B mutations in the Chinese population. In the South Korean WD population, R778L is the primary mutation with a relatively high allele frequency, but A874V is a secondary high-frequency mutation (Bae et al., 2009). Populations in Taiwan and Hong Kong, China, carry both the R778L and P992L hotspots, and their mutation distribution patterns are highly similar to those of populations in southern mainland China (Fan et al., 2023; Mak et al., 2008). Although the R778L mutation can be detected in the Japanese WD population, it accounts for only 13.4% of all pathogenic alleles; the P992 mutation is detected at an extremely low rate in Japanese cases, and there is no evidence of regional founder expansion similar to that observed in southern Chinese populations (Okada et al., 2000). Based on the above comparisons of differences among East Asian populations, it is evident that R778L is an ancient ancestral mutation shared across the broader East Asian population, whereas P992L is a unique variant that has undergone specific founder enrichment exclusively among Southern Han Chinese in mainland China, Hong Kong, Macau, and Taiwan. This cross-population comparison further strengthens the evidence supporting the divergent evolution of R778L and P992L.

### 2.2 Dual drivers of molecular evolution and enrichment

Two layers of mechanisms drive variant accumulation. At the molecular level, both mutations map to CpG dinucleotides. CpG cytosines readily undergo methylation and spontaneous deamination, rendering these sites recurrent mutational hotspots and enabling repeated *de novo* variant generation across populations (Yang et al., 2021; Li et al., 2021; Liu et al., 2020; Huster et al., 2012).

At the population genetic level, founder effects combined with weak purifying selection drive sustained variant propagation. As an autosomal recessive disorder, WD produces no overt clinical phenotype in heterozygous carriers, so pathogenic alleles experience minimal selective pressure and persist stably via genetic drift over generations. Historical population expansion and geographically isolated reproduction further elevated ancestral variant frequencies to form region-specific mutational profiles (Zhang et al., 2022; Dong et al., 2016; Yang et al., 2021).

### 2.3 Haplotype characteristics and their clinical significance

Haplotypes reflect the linkage patterns of alleles on the same chromosome. By analyzing the linkage disequilibrium between the R778L and P992L mutations and other polymorphic sites in the *ATP7B* gene, the structure of core haplotypes can be determined, providing a basis for molecular diagnosis, population tracing, and family screening (Dong et al., 2016; Yang et al., 2021). The core haplotype composed of R778L and flanking D13S315/D13S316 microsatellites shows high conservation in East Asian WD patients, which provides preliminary haplotype evidence consistent with a shared ancient ancestral origin (Bae et al., 2009), yet large-scale ancient genome data are still lacking to fully verify this inference. P992L-associated haplotypes also exhibit strong linkage disequilibrium in southern Chinese groups. These conserved haplotypes improve the sensitivity and specificity of WD genotyping and facilitate zygosity determination and genetic counselling (Yang et al., 2021; Jia et al., 2022).

Although the haplotype results observed across East Asian study cohorts are similar, current population genetics studies still have several significant limitations. First, most genetic datasets originate from single-center patient populations with limited geographic scope, resulting in significant regional and ethnic sampling biases. Second, most haplotype analyses rely on relatively small sample sizes, and few multicenter pooled datasets have been constructed, which weakens the generalizability of inferences regarding the founder effect.

## 3 Phenotypic heterogeneity: nonlinear associations between genotype and phenotype

Phenotypic variability represents a hallmark of WD among Chinese patients. Even carriers of identical *ATP7B* compound genotypes display divergent ages of onset, initial affected organs and disease progression rates, despite confirmed pathogenicity of R778L and P992L via functional assays. Genotype alone only establishes a baseline copper-metabolic defect; final clinical presentations arise from the combined effects of residual ATP7B activity, modifier genes and environmental exposures.

### 3.1 Differential association patterns based on protein functional domains

#### 3.1.1 Phenotypic characteristics of the R778L mutation

The R778L variant resides in ATP7B’s fourth transmembrane helix, the core channel mediating hepatocellular copper efflux (Liu et al., 2004; Gupta et al., 2018). Multiple Chinese clinical cohort studies have observed that R778L homozygotes present with the early-onset form, with the onset of symptoms in most cases concentrated during childhood and adolescence (Zhang et al., 2022; Xue et al., 2023a). Initial presentations often include unexplained transaminase elevation and hepatomegaly, and some cases rapidly progress to fulminant hepatic failure. A 115-patient cohort reported significantly higher alanine transaminase concentrations in R778L homozygotes relative to other genotypes (*p* = 0.024) (Xia et al., 2024). Only approximately 10%–15% of affected individuals primarily display neurological or mixed phenotypes (Zhang et al., 2022; Huster et al., 2012; Lao and Le, 2024).

#### 3.1.2 Phenotypic characteristics of the P992L mutation

The P992L mutation is in the ATP-binding catalytic domain (P-domain) of ATP7B(Gupta et al., 2018; Hua et al., 2016; Wang et al., 2024). Like carriers of the R778L mutation, most patients with the P992L mutation tend to develop symptoms at an earlier age (Zhang et al., 2022; Hua et al., 2016). It is hypothesized that partial retention of ATP7B transport capacity slows hepatic copper buildup and facilitates copper deposition in the basal ganglia (Huster et al., 2012; Gupta et al., 2018; Zhang L. et al., 2025).

**FIGURE 1:**
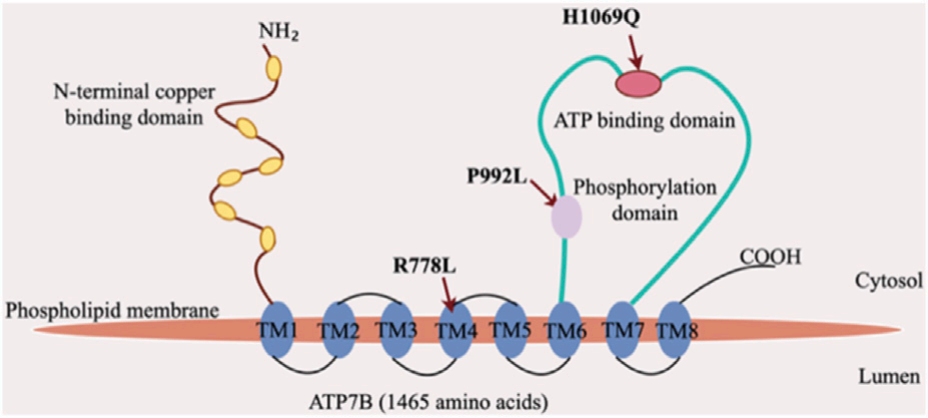
Schematic of human ATP7B domains with labeled R778L, P992L and H1069Q. Note: Four core functional regions are presented as required: N-terminal copper-binding domain, eight transmembrane helices forming the copper translocation pore, cytoplasmic phosphorylation (P) catalytic domain and ATP-binding domain. Three hotspot pathogenic variants are annotated with directional arrows: R778L locates at the 4th transmembrane helix (TM4), P992L resides within the phosphorylation catalytic domain, and H1069Q maps to the ATP-binding domain. Cytosol, cytoplasmic compartment; Lumen, Golgi luminal compartment.

The two predominant Chinese WD variants R778L and P992L, together with the common European variant H1069Q, are distributed in distinct functional regions of ATP7B, leading to divergent protein dysfunction and clinical phenotypes (Figure 1). To further distinguish their genetic, cellular and clinical discrepancies, a multidimensional cross-variant comparison is summarized in Table 1.

**TABLE 1:** Multidimensional comparative analysis of pathogenic mutations in ATP7B.

Feature	R778L	P992L	H1069Q
Variant	p.Arg778Leu	p.Pro992Leu	p.His1069Gln
Variant location	TM4 of TMD (Bitter et al., 2022)	Cytoplasmic P-domain (Bitter et al., 2022)	Cytoplasmic N-domain (Bitter et al., 2022)
Predominant population	East Asian (Chinese hotspot) (Zhang et al., 2022; Bae et al., 2009; Okada et al., 2000)	Southern Chinese (Li et al., 2019; Yang et al., 2021)	European Caucasians (Ferenci, 2006)
Population genetic	Founder haplotype, uniform across China (Yang et al., 2021; Bae et al., 2009)	Southern China founder expansion (Yang et al., 2021)	European founder effect (Ferenci, 2006)
Residual ATP7B function	Severe loss; massive ERAD degradation, nearly absent functional protein (Zhu et al., 2015)	Mild impairment; partial ATPase activity, normal Golgi localization (Zhu et al., 2015)	Slightly reduced activity, enhanced ERAD (Huster et al., 2012; Overeem et al., 2019)
Typical onset age	Early onset (Zhang et al., 2022; Xue et al., 2023a)	Early onset (Zhang et al., 2022)	Late onset (Stapelbroek et al., 2004)
Copper biochemical markers	Very low serum Cp, low serum Cu (Lu X. S. et al., 2022; Yang et al., 2022; Cheng et al., 2017)	Moderately low Cp, mild chronic Cu overload (Yang et al., 2022)	Mild Cu metabolic disturbance (Huster et al., 2012)
Core cell death pathway	Ferroptosis; dominant-negative toxicity (Zhu et al., 2015; Tang et al., 2023)	Chronic mitochondrial intrinsic apoptosis (Zhu et al., 2015)	Slow mitochondrial oxidative injury (Gromadzka et al., 2024)

TM4, 4th transmembrane helix; TMD, transmembrane domain; ERAD, Endoplasmic reticulum-associated degradation; P-domain, cytoplasmic phosphorylation catalytic domain; N-domain, N-terminal copper-binding domain; Cu, Copper; CP, ceruloplasmin.

### 3.2 The diagnostic value and variability of biochemical phenotypes

Copper-related biochemical indices form the backbone of WD diagnosis, risk stratification and therapeutic monitoring. Mutant-specific biochemical signatures partially mirror disease severity and act as intermediate readouts linking genotype and clinical phenotype (Dong et al., 2021; Zhang N. et al., 2025; Ryan et al., 2019). Despite broad phenotypic heterogeneity, consistent biomarker trends support early screening, genotypic inference and treatment surveillance.

Ceruloplasmin (CP), hepatocyte-synthesized and ATP7B-dependent, serves as a key biomarker reflecting the residual function of the ATP7B protein (Wang et al., 2024; Song et al., 2022). Large cohorts confirm universally reduced CP levels in pathogenic *ATP7B* carriers, with R778L homozygotes showing markedly lower concentrations than other genotypes (*p* < 0.05) alongside earlier disease onset and diminished serum copper (Lu X. S. et al., 2022; Yang et al., 2022; Cheng et al., 2017). In a pediatric cohort of 316 WD patients, R778L homozygotes had a mean CP of 23 ± 5 mg/L versus 61 ± 48 mg in non-mutant controls (*t* = −2.34, *p* < 0.001) (Lu Z. K. et al., 2022). Notably CP rises as an acute-phase reactant during inflammation or pregnancy, which can mask underlying deficiency (Liu et al., 2022; Inoue et al., 1999; Hellman and Gitlin, 2002).

*In vitro* functional assays demonstrate milder transport impairment for P992L: the variant does not trigger severe ER misfolding or disrupted phosphorylation intermediates, so its suppressive effect on CP synthesis is far less severe than that of transmembrane-domain mutations (Huster et al., 2012). Consistent with preclinical data, homozygous P992L patients display higher median CP (0.034 g/L, IQR 0.021–0.047) than R778L homozygotes (0.017 g/L, IQR 0.011–0.036) and other mutant genotypes (0.045 g/L, IQR 0.017–0.071) (Yang et al., 2022).

Serum free copper and 24-h urinary copper reflect systemic copper burden and track disease activity (Zeng et al., 2025; Ryan et al., 2019; Gromadzka et al., 2023a; Zhou et al., 2022). Quantitative genotype-specific correlations between these markers and R778L/P992L status remain insufficiently characterized and require further clinical validation.

## 4 The depth of phenotypic regulation: from single genes to multifactorial interactions

Identical *ATP7B* pathogenic variants yield highly variable clinical manifestations, indicating WD is a complex multifactorial trait rather than a simple monogenic disorder. Phenotype is jointly modulated by the causal gene, modifier loci, epigenetic programming and external environmental cues, forming a multilayer regulatory network that generates heterogeneous disease outcomes and supplies targets for novel therapeutic development (Dev and Hamilton, 2021).

### 4.1 The regulatory role of epigenetic modifications

Epigenetic marks alter gene expression without modifying underlying DNA sequences; major forms include DNA methylation, histone modifications and non-coding RNA networks, all of which modulate *ATP7B* activity to drive WD phenotypic divergence (Shribman et al., 2021; Gromadzka et al., 2024; Bernstein et al., 2007).

Promoter methylation is the most thoroughly characterized epigenetic modifier of *ATP7B*, directly governing its transcriptional output (Gromadzka et al., 2023b). At the same time, DNA methylation levels are susceptible to environmental factors, further increasing phenotypic heterogeneity. Modifications of histones, such as acetylation and methylation, can influence chromatin density and contribute to the transcriptional regulation of *ATP7B*(Shribman et al., 2021; Sarode et al., 2021). HDAC inhibitor treatments modulate cellular *ATP7B* mRNA levels, implying interindividual histone profiles alter residual copper transport capacity and systemic copper tolerance (Sarode et al., 2021; Gupta et al., 2011). To date, no head-to-head multi-omics studies have compared epigenetic landscapes between R778L and P992L carriers, leaving mutation-specific epigenetic cascades unvalidated.

### 4.2 Compensatory and exacerbating effects of modifier genes

Modifier genes do not independently cause WD but tune copper metabolism and oxidative stress pathways to either alleviate or worsen organ injury in variant carriers (Shribman et al., 2021; Medici and Weiss, 2017; Zhou et al., 2025).

*ATOX1* encodes a cytoplasmic copper chaperone that shuttles copper to ATP7B, with its functional status directly determining mutant protein transport efficiency (Uhlemann et al., 2020; Yu et al., 2017). The protein encoded by the *COMMD1* gene is a key factor in regulating the transport of ATP7B from the Golgi apparatus to the cell membrane and can influence the efficiency of copper excretion in bile (Zhou et al., 2025; Materia et al., 2012). COMMD1 does not directly regulate the copper-induced ATP7B transport process, but it can enhance the clearance of misfolded ATP7B via the ERAD pathway by abnormally binding to mutant ATP7B, thereby reducing levels of functional ATP7B and exacerbating copper accumulation (de Bie et al., 2007; Fedoseienko et al., 2014). *MTHFR* rs1801133 variants modulate oxidative stress via homocysteine metabolism. TT homozygotes display elevated homocysteine, heightened oxidative damage and severe hepatic/neuronal lesions, whereas CC genotypes confer milder disease with reduced oxidative burden (Gromadzka et al., 2011; Ferenci, 2011).

### 4.3 Interaction driven by environmental exposure

External exposures cannot initiate WD but interact with genotype, epigenetics and modifier genes to amplify phenotypic heterogeneity. Key modulating factors include dietary copper intake, drinking water copper concentration and lifestyle patterns (Shribman et al., 2021; Gromadzka et al., 2023b; Teufel-Schäfer et al., 2022). Sustained high-copper diets or contaminated water exceed physiological copper clearance thresholds and accelerate symptomatic onset (Dong and Wu, 2012; Wang et al., 2026), an effect more variable for variants like P992L that retain partial transport function. Additional stressors including chronic alcohol overconsumption, persistent fatigue, childhood infections and certain pharmaceuticals exacerbate hepatic metabolic load and oxidative injury, acting as clinical triggers, thereby influencing the clinical phenotype.

It is worth noting that current evidence regarding epigenetic modifiers, MTHFR polymorphisms, and the ceRNA regulatory axis all stems from small-scale *in vitro* cell experiments or rodent animal models; there is currently a lack of prospective, multi-omics validation studies in humans targeting stratified R778L/P992L patient subgroups. The collection of patient samples from a single source and the limited sample size have hindered comparisons across subgroups, resulting in a lack of reproducible results among independent research teams.

### 4.4 Critical limitation of existing mechanistic evidence

All published data on epigenetic regulators, *MTHFR* variants and ceRNA axes derive from small *in vitro* cell assays or rodent models. Large prospective human multi-omics studies stratified by R778L/P992L genotypes remain absent. Single-source patient cohorts and limited subgroup sample sizes restrict cross-study comparability and reproducibility of these regulatory mechanisms.

## 5 Molecular mechanisms: from protein dysfunction to changes in cell fate

R778L and P992L both triggers disrupted copper trafficking and intracellular metal overload, yet distinct mutational locations and protein defects drive divergent rates of copper buildup and divergent cell death cascades.

### 5.1 Site-specific structural and functional defects of ATP7B

The phenotypic differences caused by the R778L and P992L mutations essentially stem from variations in the mutation sites, which result in differences in the extent of structural, intracellular transport, and functional impairments of the ATP7B protein, thereby leading to distinct rates of copper accumulation and patterns of cellular damage (Huster et al., 2012; Zhu et al., 2015; Tang et al., 2023). *In vitro* experiments have demonstrated that, under conditions of copper overload, less than 10% of the R778L mutant protein is transported to the bile duct membrane, whereas more than 80% of the wild-type protein is transported (Tang et al., 2023; Wei R. et al., 2022). Another *in vitro* functional assay showed that the copper transport activity of the R778L mutant was only 30%–40% that of the wild type (Huster et al., 2012). This mutation results in the near-complete blockage of the bile-mediated copper efflux pathway, leading to the rapid and massive accumulation of copper ions within liver cells. Misfolded R778L protein forms aggregates within cells, exerting a dominant-negative effect that may further exacerbates copper transport defects. It also induces severe mitochondrial damage and oxidative stress, and mediates copper-dependent ferroptosis, which may act as a major contributor of early-onset liver damage in patients with this mutation (Shribman et al., 2021; Christianson and Carvalho, 2022; Kim et al., 2020).

The P992L mutation is in the ATP-binding catalytic domain of ATP7B, primarily resulting in reduced ATPase activity and insufficient energy supply (Huster et al., 2012; Zhu et al., 2015; Kim et al., 2020). The mutated protein is initially localized to the Golgi apparatus; however, due to reduced ATP hydrolysis efficiency and insufficient energy supply, it cannot be effectively transported to the bile duct membrane, ultimately leading to a gradual decline in the bile’s ability to excrete copper. The P992L mutation has a relatively minor impact on protein folding, allowing the protein to retain some basic functions. Copper accumulation occurs at a relatively slow rate, primarily leading to chronic mitochondrial damage and apoptosis; consequently, patients with this mutation exhibit a higher prevalence of the neurological and mixed phenotypes (Zhang et al., 2022; Zhang L. et al., 2025).

### 5.2 Protein quality control and localization disorders: a key mechanism of functional inactivation

The functional defects in ATP7B mutants are not solely due to amino acid substitutions; the cellular protein regulatory system’s role in clearing misfolded proteins also plays a critical part (Huster et al., 2012; Tang et al., 2023). The R778L mutation replaces a charged arginine in the transmembrane domain with a hydrophobic leucine, altering the insertion angle and structural stability of the transmembrane segment, thereby triggering the endoplasmic reticulum-associated degradation (ERAD) pathway (Wei R. et al., 2022). The mislocalized R778L protein is recognized by the endoplasmic reticulum and degraded via the ubiquitination pathway; only a small fraction of the protein is transported to the distal Golgi network. Additionally, because the transmembrane channel structure is disrupted, this mutant protein is unable to respond to copper signals and complete its transport to the bile duct membrane (Christianson and Carvalho, 2022; Kim et al., 2020). This dual defect—characterized by insufficient protein synthesis and a complete loss of transport function—further explains why the R778L mutation is prone to inducing early-onset severe liver injury.

In contrast, the P992L mutant protein has a relatively normal folding conformation and can partially evade ER-mediated degradation; its pathogenic mechanism lies in a significant reduction in ATPase activity (Huster et al., 2012; Kim et al., 2020). Although this mutant protein can be transported to the distal end of the Golgi apparatus, it is unable to efficiently transport copper due to insufficient energy supply, resulting in persistently low copper efflux. This finding is highly consistent with the clinical characteristics of this patient group, which include slow copper accumulation, late-onset disease, and a relatively mild course.

**FIGURE 2:**
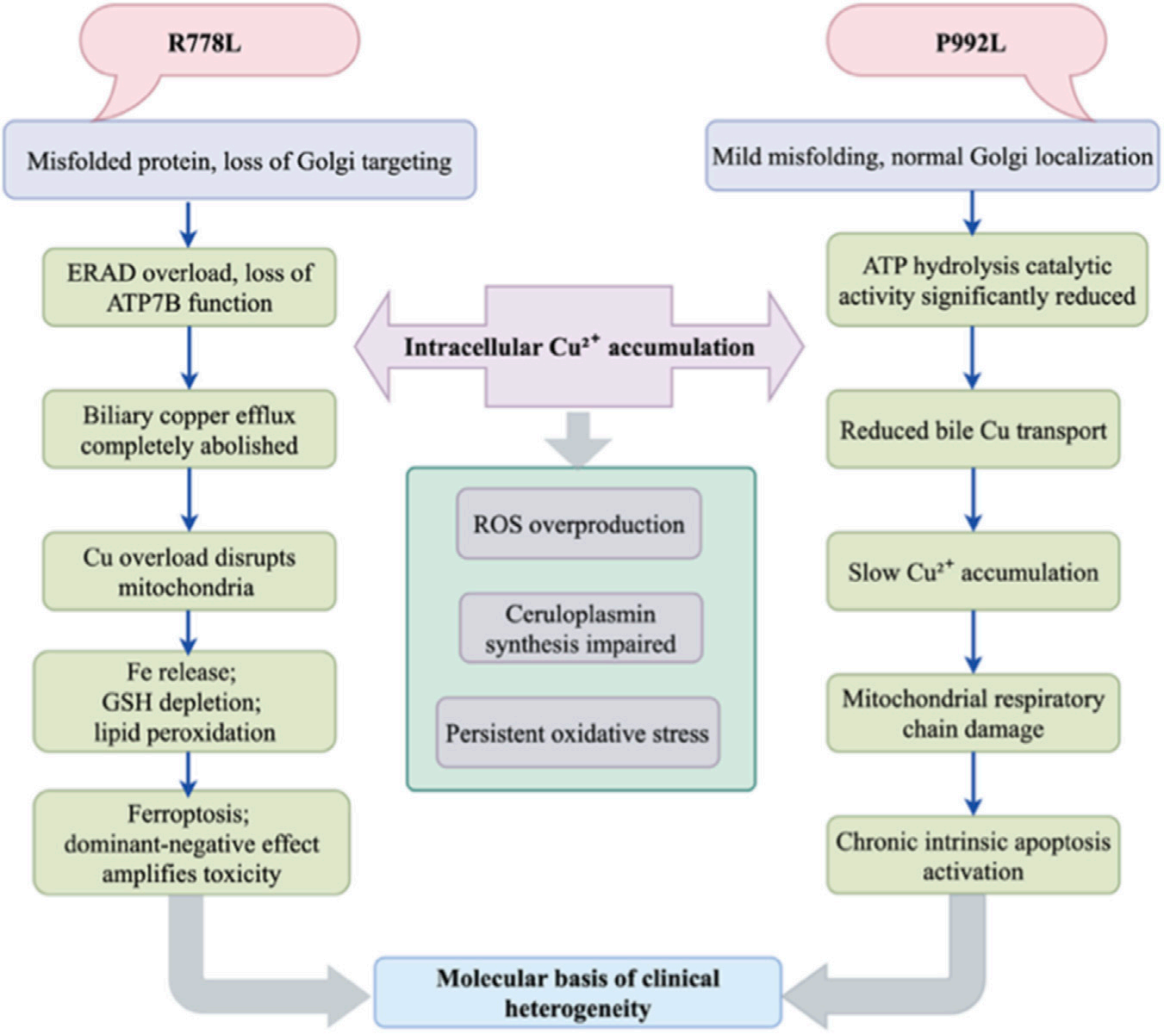
Comparative signaling flowchart of intracellular copper toxicity induced by ATP7B R778L and P992L variants.

The two predominant Chinese WD variants R778L and P992L reside in distinct ATP7B functional domains and trigger completely divergent intracellular copper injury cascades, which fundamentally explain the prominent clinical heterogeneity among Chinese WD patients (Figure 2).

### 5.3 Cellular damage cascades: mitochondrial toxicity and ferroptosis

Excessive levels of free copper in the cytoplasm can activate multiple cell death pathways (Xue et al., 2023b; Tsvetkov et al., 2022; Wan et al., 2020). Copper influx into mitochondria damages mtDNA and respiratory complexes, suppressing ATP synthesis and triggering oxidative stress (Shribman et al., 2021). In the context of the R778L mutation, bile copper excretion is nearly completely blocked, and mitochondrial damage occurs acutely and explosively, representing a key mechanism underlying early-onset severe liver injury (Huster et al., 2012). Recent studies have shown that copper overload can induce ferroptosis in hepatocytes by exacerbating lipid peroxidation and disrupting the homeostasis of cellular iron metabolism (Tang et al., 2023; Xue et al., 2023c), thereby providing a new molecular mechanism for acute hepatic necrosis in WD. Ferroptosis likely represents a shared late-stage toxic endpoint for copper overload, yet its relative contribution to variant-specific phenotypic differences requires large cohort validation.

### 5.4 Molecular regulatory networks: the modulatory role of non-coding RNAs

Non-coding RNAs modulate copper-metabolic protein expression and mutant ATP7B stability, forming an epigenetic layer driving variable WD presentations (Sarkar et al., 2025; Wei T. H. et al., 2022). In colorectal cancer models, circHN1 acts as a molecular sponge to sequester miR-302a-3p and relieve its inhibitory effect on ATP7B transcription (Wu et al., 2022). Whether the dysregulation of this regulatory axis exacerbates impaired copper excretion in WD patients with copper overload and increases phenotypic heterogeneity requires further support from cellular experiments or clinical data.

Liver-specific miR-122-5p targets copper transport, oxidative stress and lipid metabolism mediators, correlating closely with hepatic inflammation and fibrosis severity in WD (Sánchez-Monteagudo et al., 2024). LncRNAs including H19, MALAT1 and MEG3 interact with miRNAs to indirectly tune ATP7B and copper-homeostasis gene expression (Zhang et al., 2021), so interindividual non-coding RNA levels partially account for phenotypic divergence among patients carrying identical ATP7B variants.

## 6 Targeted intervention strategies: therapeutic approaches and future directions based on mutation profiles in the Chinese population

Given the mutation profiles of WD patients in China, which are dominated by the R778L and P992L mutations, mutation-specific targeted interventions have emerged as a key focus in precision therapy. Current therapeutic approaches fall into two distinct categories: well-established routine clinical treatments, and preclinical mutation-targeted experimental strategies still unvalidated in human trials. Standard copper-chelation regimens form the clinical mainstay, while molecular chaperones and gene editing remain confined to cell and animal research with no mature clinical application.

### 6.1 Established clinical copper-lowering therapies

First-line standard treatments rely on chelators and zinc supplements. Differential therapeutic responses between R778L and P992L carriers, highlighting the value of genotype-personalized dosing schemes. The R778L mutation causes disruption of the transmembrane domain, abnormal protein folding, and enhanced ERAD, resulting in near-complete obstruction of bile-mediated copper excretion. This leads to rapid copper accumulation and high dependence on chelating agents. However, due to the near-complete loss of ATP7B function, monotherapy with chelating agents often fails to rapidly reverse severe acute liver injury; therefore, combination therapy with organ protection and intensive supportive care is required (Huster et al., 2012; Tang et al., 2023; van den Berghe et al., 2009). For irreversible fulminant hepatic failure or decompensated cirrhosis, liver transplantation serves as the definitive life-saving intervention, as it restores intact biliary copper clearance and resolves systemic metal overload long-term. Its widespread use is limited by organ shortage, rejection risk and lifelong immunosuppression requirements, so it cannot be adopted for early disease management. Clinical evidence indicates that in patients with predominantly neurological ATP7B mutations, the initial phase of high-dose chelator therapy may induce a transient exacerbation of neurological symptoms due to marked fluctuations in serum free copper levels and copper redistribution effects. This phenomenon exhibits a clear genotype-specific correlation in patients with the neurological subtype of WD (Antos et al., 2023), The mechanism involves rapid copper mobilization, which causes the liver to release large amounts of copper into the bloodstream. Once copper ions cross the blood-brain barrier, they exacerbate oxidative stress and neuronal damage in the brain, leading to a short-term worsening of symptoms such as motor dysfunction and dysphagia (Shribman et al., 2021; Litwin et al., 2024). Neurological WD patients therefore require slow dose titration starting at low baseline levels. Ammonium tetrathiomolybdate (ATTM) stands out for this subgroup: unlike d-penicillamine and trientine, which provoke harmful copper redistribution, ATTM forms stable copper complexes in the gut and peripheral circulation, limiting cerebral copper delivery and lowering the risk of neurological flare-ups (Stremmel, 2019; De Fabregues et al., 2020).

### 6.2 Preclinical mutation-targeted experimental therapies

Cell and animal studies have shown that pharmacological chaperone molecules such as 4-phenylbutyric acid and curcumin may reduce ERAD-mediated degradation by promoting the correct folding of the R778L mutant protein and restoring partial transport function (van den Berghe et al., 2009; Zhang et al., 2011), suggesting that such molecules hold potential for targeted therapies against folding-defect mutations such as R778L. Laboratory evidence suggests that copper overload induces ferroptosis in hepatocytes through lipid peroxidation and iron metabolism disorders, thereby exacerbating acute liver injury; ferroptosis inhibitors may provide adjunctive protection during the acute phase of liver injury (Zhao et al., 2024; Li et al., 2020). However, research is currently limited to basic and translational studies, and clinical safety, timing of administration, and the risk-benefit ratio still require validation through clinical trials.

The combination of CRISPR-Cas9 and single-stranded oligonucleotides enables precise repair of ATP7B mutations in HEK293T cells, laying a critical methodological foundation for WD gene editing therapy (Pöhler et al., 2020). Reprogrammed hepatocytes engineered with mini-ATP7B recover copper-dependent trafficking and resist oxidative damage *in vitro*, and mouse transplantation models demonstrate alleviated hepatic copper deposition and fibrosis (Cai et al., 2022). Building on this, the study achieved heterozygous gene correction in induced pluripotent stem cells derived from WD patients carrying the R778L homozygous mutation using a combination of CRISPR/Cas9 and single-stranded oligodeoxynucleotides (Wei R. et al., 2022). The hepatocyte-like cells differentiated from these cells not only restored the subcellular localization, copper-responsive transport, and copper efflux functions of ATP7B *in vitro*, but also, following *in vivo* transplantation, significantly reduced hepatic copper accumulation, copper-induced hepatotoxicity, and liver fibrosis in immunodeficient WD model mice, providing core proof-of-concept validation for the clinical application of autologous cell therapy for WD.

In the field of virus-mediated gene replacement therapy, AAV-vector-mediated ATP7B mini-gene therapy has become a central area of research in WD gene therapy. Pharmacokinetic modeling results for VTX-801 indicate that its efficacy in restoring bile copper excretion, reducing hepatic copper load, and rebalancing copper metabolism is dose-dependent (Murillo et al., 2022; Lindauer et al., 2026). In basic research, intravenous infusion of the codon-optimized human ATP7B gene delivered via an AAV8 vector in WD mice with early-stage disease resulted in a dose-dependent reduction in hepatic copper accumulation and alleviation of liver damage; high-dose intervention was even able to completely prevent the onset and progression of liver fibrosis (Greig et al., 2019). As early as 2012, a preclinical animal study conducted delivered the human ATP7B gene to fetuses of WD model mice via intrauterine injection during early pregnancy (Roybal et al., 2012). This intervention achieved long-term, stable expression of the target protein in the liver, markedly ameliorated liver pathology, restored copper metabolic homeostasis and rescued liver function. Chinese teams have developed truncated ATP7B constructs optimized for AAV packaging; liver-targeted AAV8 delivery produces long-term copper correction in murine WD models (Leng et al., 2019).

A core limitation of universal wild-type AAV gene supplementation is its lack of specificity for China’s predominant R778L and P992L variants. *In situ* CRISPR editing directly repairs endogenous pathogenic alleles, making it the priority direction for future curative personalized WD therapy. All mature clinical regimens and mutation-specific preclinical experimental interventions for Chinese WD patients are systematically categorized in Table 2.

**TABLE 2:** Clinical and preclinical treatment strategies for Wilson disease.

Intervention	Core mechanism	Advantages and clinical limitations	Research stage
zinc supplements	Block intestinal Cu absorption, reduces systemic Cu load (Schilsky et al., 2023; Piotr et al., 2025)	Low AEs; slow Cu clearance onset, not for severe acute overload (Schilsky et al., 2023)	First-line maintenance
Oral chelators (D-penicillamine, etc.)	Bind free Cu, promotes urinary excretion (Członkowska et al., 2018; Schilsky et al., 2023)	Long-term Cu control; high-dose initial risk of neurological worsening via Cu redistribution (Antos et al., 2023)	First-line chelation
Ammonium tetrathiomolybdate	Forms stable Cu complexes in gut/blood, prevents Cu crossing BBB (Stremmel, 2019)	Minimizes transient neurological worsening; limited Chinese clinical experience (Xie and Wu, 2017)	Second-line rescue
Orthotopic liver transplantation	Restores physiological biliary Cu excretion (Schilsky et al., 2023)	Radical correction; limited by organ shortage, surgical risk, lifelong immunosuppression (Piotr et al., 2025)	Curative for end-stage liver disease
Pharmacological chaperones (4-PBA, etc.)	Correct misfolded ATP7B, reduces ERAD, restores partial Cu transport (Materia et al., 2012; van den Berghe et al., 2009)	Targets root folding defect (R778L); no long-term human safety data (van den Berghe et al., 2009; Zhang et al., 2011)	preclinical
Ferroptosis inhibitors	Suppress lipid peroxidation and iron-mediated hepatotoxicity from rapid Cu overload (Xue et al., 2023c; Li et al., 2020)	Alleviates acute ferroptotic damage; efficacy/window unvalidated in humans (Zhao et al., 2024; Li et al., 2020)	Preclinical
AAV-ATP7B mini-gene therapy	Liver-targeted delivery of functional ATP7B to rebuild biliary Cu clearance (Leng et al., 2019)	Durable hepatic ATP7 expression, dose-dependent correction (Murillo et al., 2022); cannot repair endogenous alleles	Preclinical mouse model; early translational
CRISPR-Cas9/ssODN	Directly correct pathogenic point mutations (Pöhler et al., 2020)	Permanent genotype correction; off-target risks unresolved (Pöhler et al., 2020)	iPSC and preclinical animal stage

Cu, Copper; BBB, blood-brain barrier; 4-PBA, 4-phenylbutyrate; ERAD, endoplasmic reticulum-associated; AAV, adeno-associated virus; ssODN, single-stranded oligodeoxynucleotide; iPSC, induced pluripotent stem cells; AEs, adverse events.

## 7 Concluding remarks

Chinese WD is uniquely defined by two dominant pathogenic variants, R778L and P992L. Their population enrichment arises from combined CpG mutational hotspots and founder expansion, supported by highly conserved East Asian haplotypes that underpin region-specific molecular screening frameworks. Distinct structural defects of the two variants drive divergent clinical presentations. Multilayered regulatory crosstalk among mutant *ATP7B*, epigenetic marks, modifier loci and environmental copper exposure collectively explains the broad phenotypic variability observed among carriers of identical hotspot genotypes.

Despite accumulating mechanistic findings, substantial translational barriers persist, chiefly stemming from uneven evidence quality graded by study design. High-grade evidence: Large prospective multicenter natural history cohorts tracking R778L/P992L carriers remain extremely scarce in China. Moderate limited evidence: Retrospective single-center hospital cohorts dominate existing clinical data, constrained by small sample sizes, unbalanced regional/ethnic recruitment, selection bias and publication bias that limit national generalizability. Low-grade preclinical evidence: Cell and rodent assays only supply tentative mechanistic clues, without human validation. Most current clinical inferences rest on moderate or low-quality datasets and cannot act as definitive predictive benchmarks. Critically, mutation-targeted precision modalities (molecular chaperones, gene editing) remain confined to preclinical testing with no long-term human safety or efficacy data, clearly distinguishable from well-established routine copper-chelation regimens applied in daily clinical practice.

Taken together, this review delineates the unique genetic and molecular landscape of Chinese WD while highlighting urgent gaps requiring cohort construction, multi-omics validation and translational research to advance localized genotype-guided disease management.

## 8 Future perspectives

Four core research directions are proposed to bridge laboratory discoveries and real-world WD care tailored to Chinese populations. First, construct nationwide multicenter cohorts and integrated biobanks covering full disease trajectories of R778L and P992L carriers, to power genotype-stratified analyses and identify actionable modifier genes. Second, deploy multi-omics profiling to map dynamic copper-toxicity signaling cascades and develop robust predictive biomarkers for disease progression. Third, optimize mutation-adapted preclinical interventions: refine hepatotropic AAV delivery systems and CRISPR editing platforms alongside folding-correcting small-molecule chaperones, to accelerate translational research. Fourth, build a complete lifelong WD management system. Given China’s higher overall WD prevalence and elevated carrier frequencies of the two local hotspots, newborn screening workflows should be optimized by pairing biochemical ceruloplasmin screening with targeted *ATP7B* variant genotyping. Haplotype-based testing can expand early detection to asymptomatic neonates and at-risk relatives, forming an integrated system covering neonatal screening, genetic counselling, standardized clinical intervention and long-term primary care follow-up.

Focused research centred on R778L and P992L establishes a foundational framework for China’s WD precision medicine pipeline. With deeper mechanistic characterization and translational breakthroughs in variant-specific therapeutics, clinical management will shift from empirical lifelong copper chelation to mechanism-driven targeted correction. Cross-disciplinary collaborative efforts will further elevate domestic WD care standards, enabling comprehensive prevention, early diagnosis, individualized treatment and sustained long-term disease control.
